# Identifying key determinants influencing the sustainment of physical activity and nutrition programs in Australian primary schools

**DOI:** 10.1186/s12966-025-01808-6

**Published:** 2025-08-30

**Authors:** Edward Riley-Gibson, Alix Hall, Adam Shoesmith, Rachel C. Shelton, Christophe Lecathelinais, Rebecca K. Hodder, Luke Wolfenden, William Pascoe, Carly Gardner, Kate M. O’Brien, Emma Pollock, Rachel Sutherland, Nicole Nathan

**Affiliations:** 1 School of Medicine and Public Health, The University of Newcastle, Newcastle, NSW Australia; 2 The National Centre of Implementation Science (NCOIS), The University of Newcastle, Newcastle, NSW Australia; 3 Hunter Medical Research Institute, New Lambton Heights, Newcastle, NSW Australia; 4 Hunter New England Population Health, Hunter New England Local Health District, Newcastle, NSW Australia; 5 Department of Sociomedical Sciences, Columbia University, Mailman School of Public Health, New York, NY Australia

**Keywords:** Implementation science, Sustainment, Sustainability, Chronic disease prevention, Sustainability determinants, Physical activity, Nutrition, Evidence based intervention

## Abstract

**Background:**

To ensure the large number of school-based physical activity and nutrition programs have a lasting positive impact on the health and wellbeing of students, it is essential that such programs are sustained long-term. However, there is limited research assessing the duration of such programs and the determinants that are related to their sustainment. This study investigates the duration of, and determinants to the sustainment of physical activity and nutrition programs in Australian primary schools.

**Methods:**

A cross-sectional study with 207 Principals (one from each school) from a nationally representative sample of randomly selected Australian primary schools. Principals completed a survey online or via telephone, which included items assessing the determinants of program sustainment categorised based on the domains of the Integrated Sustainability Framework (inner contextual factors; outer contextual factors; characteristics of the intervention; and processes). Schools were randomised to answer survey items relating to either physical activity or nutrition programs. We collected data on the number and type of programs, their duration, and factors influencing the sustainment of one selected program. Descriptive statistics were used to assess the duration and prevalence of programs. Regression analysis was used to assess the association between sustainment determinants and the duration of program delivery.

**Results:**

Schools randomised to physical activity programs implemented on average, 5.4 of the nine physical activity programs assessed. Schools randomised to nutrition implemented on average, 2.8 of the seven nutrition programs assessed. Physical activity programs had a mean duration of 6.9 years and nutrition programs had 7.4 years. Nutrition programs had 3.27 times the odds of being sustained longer than physical activity programs (95% CI: 1.57, 6.83; *p* = 0.002). The only domain from the Integrated Sustainability Framework that was statistically significantly associated with the sustainment for both physical activity and nutrition programs was outer contextual factors. This domain includes the alignment of the program with the priorities of the school, partnerships between the school and external organisations, and the existence of a governing body policy or guideline related to the program. The highest ranked determinant from this domain for both physical activity and nutrition programs was the alignment of the program with the priorities of the school.

**Conclusion:**

This study highlights the need for targeted strategies to support the sustainment of health programs in schools, particularly focusing on outer contextual factors. Specifically, the alignment of the program with the priorities of the school. Policymakers and practitioners should prioritise targeting these outer contextual determinants to enhance the sustainment of physical activity and nutrition programs, ultimately promoting better long-term population health outcomes.

**Supplementary Information:**

The online version contains supplementary material available at 10.1186/s12966-025-01808-6.

## Background

Chronic diseases such as cardiovascular disease, type 2 diabetes and some cancers are among the leading causes of death and disability globally [1]. Poor dietary habits and insufficient physical activity are leading modifiable risk factors for such chronic diseases, with 5.7 and 7.9 million non-communicable disease related deaths attributable to poor diet and physical inactivity respectively [1, 2]. As these health behaviours often develop in childhood and track into adulthood [3], promoting healthy lifestyles among children is a recommended global public health strategy [4–6].

Schools are a key setting for targeting physical activity and healthy eating in children as they: i) provide access to the majority of children aged 5–18 years [7] for extensive periods (approximately 30 h per week) of time during crucial developmental stages [5, 6, 8]; and ii) are well-resourced settings that enable the delivery of priority areas and policy guidelines supporting healthy behaviours in children and youth [5, 6, 8]. Evidence suggests school-based interventions, practices, programs, and innovations (hereby collectively referred to as programs) that increase children’s opportunities to be physically active during the school day e.g., through physical education, sport or short classroom physical activity breaks such as energisers, are effective in increasing children’s moderate to vigorous physical activity [5, 9, 10]. Similarly, interventions aimed at improving children’s nutrition in schools, for example through fruit and vegetable breaks, free fruit and vegetables provided to students, and healthy lunch box initiatives, have also been found to be effective [11–13].

To maximise the public health impact of such effective programs, they must be successfully implemented, and their delivery sustained over time [14–16]. Sustainment is defined as “*the extent the innovation is in place or being delivered long-term”* [17]. The sustainment of public health programs is however a significant challenge. A systematic review on the sustainment of public health and clinical interventions, found that of the 125 included, only 23% of programs were sustained at least 24 months following cessation of initial implementation support [18]. Specifically, in the school setting, a recent systematic review of the sustainability of public health programs in schools found that of the 18 included programs, none continued to be delivered in their entirety (i.e., all components) following the withdrawal of initial start-up funding and provision of resources [19]. The failure to sustain effective programs is a significant concern as it: i) wastes the considerable health system investment required to achieve initial implementation [14]; ii) often results in the prevalence of health issues reverting to pre-intervention levels or worse [18–20]; iii) diminishes community trust and confidence in the benefit of such initiatives [16]; and iv) raises ethical concerns regarding end-user participation in future public health initiatives [14].

These challenges underscore the critical need for comprehensive assessments of the sustainment of physical activity and nutrition programs in schools. Despite the significant investment in such programs, there has been limited research evaluating their sustained delivery [21]. The United States’ School Health Policies and Practices Study (SHPPS) conducted national surveys between 1994—2016 to assess school health promotion policies and practices at the state, district, school, and classroom levels [22]. Whilst this provided valuable insights into the prevalence of these programs, it did not track their sustained delivery over time. Similarly, a longitudinal study of approximately 400 Australian primary schools’ adoption of healthy eating and physical activity policies and programs conducted between 2006 and 2013 found notable increases in the adoption of healthy eating programs and mixed trends in the adoption of physical activity programs [23]. However, there is an absence of information regarding the prevalence of the sustained delivery of physical activity and nutrition programs in schools, and whether the prevalence and duration varies between physical activity and nutrition programs. Understanding the prevalence of sustainment is important because it provides essential information on whether these programs continue to be implemented over time and for how long. This insight is crucial to determine if and when additional support may be required to ensure that the delivery of these programs is sustained. Moreover, assessing whether there is a difference in the sustainment of physical activity and nutrition programs can identify specific areas that may need more focused support. Knowing whether one type of program has greater difficulty sustaining than the other will help determine where efforts are needed to support sustainment.

To develop targeted strategies to effectively support the on-going delivery of programs, it is essential to identify the determinants which impact their sustainment [24]. However, there is a lack of empirical testing of which factors predict or are associated with sustainment indicators among large/robust samples. While systematic reviews have identified a range of determinants influential to the sustainability of school based public health interventions such as the availability of facilities or equipment, continued executive or leadership support, and workforce turnover [14, 19, 25], they often lack an in-depth exploration of the nuances involved in sustaining physical activity and nutrition programs. Thus, further investigation is needed to understand if these determinants differ depending on the type of program being delivered. In addition, these reviews emphasised the need for studies that use theoretically informed measures of sustainability determinants. Such measures will help accurately identify the most influential factors for sustaining school-based physical activity and nutrition programs. Further, developing an understanding of both the broad areas where strategies may need to be focused (domain level), and the specific areas within these domains that need to be targeted (item level), will guide the development of targeted strategies to address the primary determinants specific to each program type, thereby enhancing program sustainment. [14, 19, 25].

This study seeks to address the following critical evidence gaps by:

1. Evaluating the prevalence and sustained delivery of physical activity and nutrition programs in Australian primary schools.
2. Identifying differences in program delivery duration between physical activity and nutrition programs.
3. Identifying domain level determinants associated with delivery duration sustainment (sustainment indicators).
4. Identifying the top reported item level determinants associated with program delivery duration from domains significantly associated with delivery duration (sustainment).

## Methods

### Ethical approval

Ethical approval to conduct the study was received from the University of Newcastle Human Research Ethics Committee (approval no. H-2021–0045) and 22 school jurisdictions across Australia (appendix 2).

### Study design and setting

A cross-sectional study was conducted using a nationally representative randomised sample of Australian primary school principals from all Australian states and territories.

### Sample

Australian schools from government, Catholic, and independent sectors with primary school enrolments were eligible for participation. Proportional stratified random sampling was used by the Australian Centre for Education Research (ACER) to select schools. Schools were stratified according to state/territory, education sector, rurality (very remote, remote, outer regional, inner regional, major cities), Socioeconomic status (using Socio-Economic Indexes for Areas (SEIFA) Index of Education (IEO) National Decile) and school size (total enrolment quartiles). Special purpose schools such as hospital schools, sport schools, and schools that cater exclusively for children with disabilities were excluded from the study.

### Recruitment and data collection procedures

Principals of all eligible schools were sent an information pack to their publicly available school email address (via Research Electronic Data Capture [REDCap]) [26], which included an information statement outlining the purpose of the study and inviting them (or a nominated representative from their school executive) to participate and complete the survey. The pack included an online consent form with options to complete the survey online or via a Computer Assisted Telephone Interview (CATI). Principals who elected to complete the survey via CATI were telephoned by a trained research assistant who recorded participant responses within REDCap. CATI surveys took approximately 30 min to complete. Participants who elected to complete the online survey were emailed a unique link to a REDCap survey which took approximately 30 min to complete. Data were collected from August 2022 to October 2023.

### Measures

#### School characteristics

School characteristics including jurisdiction type (state/territory), school sector (government, Catholic, independent), school size (number of enrolments), socio-economic status (SES) and rurality (determined by postcode) were provided by ACER. The principal survey included items regarding school year levels and the presence and usage of the school canteen.

#### Participant characteristics

Principals or their nominated executive (e.g., deputy principal, assistant principal, member of school executive or school leader) were asked to specify their current role and how long they have held that position.

#### Schools' implementation of physical activity and nutrition programs

To avoid participant burden, schools were pre-randomised to answer questions regarding physical activity programs or nutrition programs in their school. Participants were asked if they were currently implementing a range of physical activity or nutrition programs (sourced from global syntheses of school-based studies) [11, 12, 27, 28], using response options ‘yes’, ‘no’, or ‘unsure’. Physical activity program options included: trackers to monitor students’ physical activity, teacher-led physical activity during recess and lunch breaks, student-led physical activity at recess and lunch, standing desks, allowing students to wear physical activity-enabling uniforms every day, environments that support physical activity, short physical activity class breaks, and programs to increase the quality of physical education classes and physical activity within other key learning areas. Nutrition programs included: free fruit and vegetables, food growing experiences, incentives for choosing healthy food, cooking program(s) with parents, school breakfast programs, healthy eating strategies for canteens, and fruit and vegetable breaks. Participants were asked additional follow-up questions pertaining to the sustainment of one selected program, including the duration (in months and years) of program delivery and potential determinants to its sustained delivery (see below for questions on determinants).

#### Determinants of program sustainment

Using the same selected program, participants were asked 28 items relating to the hypothesised determinants of program sustainment (see Additional file 1). Items were developed based on four domains of the Integrated Sustainability Framework (outer-contextual factors; inner-contextual factors; processes; and characteristics of the intervention), which is an empirically-informed sustainability determinants framework [16]. The Integrated Sustainability Framework was selected as it: (i) highlights key determinants that the emerging evidence suggests are important for facilitating intervention sustainment across a range of types of settings, including schools; (ii) helps to identify and organise determinants that may be important in facilitating sustainment of a program; and (iii) provides clear definitions for how determinants can be categorised into framework domains [16]. The scale items were iteratively developed with experts in implementation science, sustainability, education, and measure development, and were piloted with members of the target population, based on recommended procedures for rigorous measure development [29, 30]. As a result of item development processes and assessment of face validity and content validity, factors relating to the characteristics of the interventionist and population domain were deemed inappropriate to be answered by school executives because these aspects are outside of their role and responsibilities. As such, this domain was removed from the measure. The final measure domains included ‘outer-contextual factors’ (3 items), ‘inner-contextual factors’ (8 items), ‘processes’ (4 items), and ‘characteristics of the intervention’ (13 items) (see Additional file 1. for the survey items). Cronbach’s alpha was calculated for each domain to assess the internal consistency of the scale. Inner contextual factors, processes, and characteristics of the intervention each showed ‘good’ internal consistency, according to the psychometric and pragmatic evidence rating scale (PAPERS) for assessing internal consistency [30, 31] (inner contextual factors, α = 0.85; processes, α = 0.86; characteristics of the intervention, α = 0.87). Outer contextual factors had the lowest Cronbach’s alpha (α = 0.49). Using the physical activity or nutrition program that was selected, school executives were asked to indicate the degree to which they perceived each factor to influence sustained delivery of their program using a four-point Likert scale: ‘not at all influential’, ‘slightly influential’, ‘moderately influential’, ‘extremely influential’. Participants could also select ‘not applicable to me’ if they did not perceive the factor to be relevant to them.

#### Sustainment classification

Sustainment was assessed continuously as the number of months or years a program had been delivered for as well as classified into four categories suggested as meaningful by the literature: ‘not yet sustained’ (< 6 months post implementation) ‘early maintenance’ (6–12 months post implementation), ‘maintenance’ (12–24 months post implementation), and ‘sustained/ing’ (24 + months post implementation) [18, 32–34].

### Statistical analysis

#### Aim 1. Evaluating the prevalence of the sustained delivery of physical activity and nutrition programs in Australian primary schools

Descriptive statistics were used to outline principal and school characteristics, as well as the prevalence and duration of nutrition and physical activity programs implemented in schools.

#### Aim 2. Identifying differences in program delivery duration between physical activity and nutrition programs

Linear mixed regression analyses were used to compare the differences in length of program delivery by physical activity and nutrition programs. A random intercept was included for state and a fixed effect for program and each of the stratification variables (school type (government/Catholic), jurisdiction type, education sector, rurality (very remote, remote, outer regional, inner regional, major cities), SES, and school size). Mixed ordinal logistic regression analysis was conducted with state as a random intercept, to assess which program type (physical activity or nutrition) had the higher odds of being in a greater phase of sustainment (early maintenance, maintenance, sustained/ing). The assumption of proportional odds was supported by a proportional odds test; however, we were unable to adjust for potential confounders as when the confounders were included, the proportional odds assumption was not met.

#### Aim 3. Identifying domain level determinants (sustainment indicators) associated with delivery duration sustainment

The association between the four measure domains of the sustainment determinants measure (independent variable) and length of program delivery (dependent variable) was assessed using linear mixed regression analysis. A separate model was calculated for each of the domains. A random intercept for state was included, along with fixed effects for the determinant domain score and the following potential confounders. Domain scores were calculated for each of the four domains of sustainment determinants by summing item scores within a domain together and dividing by the number of non-missing items. Only participants who responded to at least half of the items in a domain were calculated a domain score. The association between the categories of program length of delivery and domain scores was assessed using ordinal mixed regression analysis which included a random intercept for state and fixed effects for the domain score but did not control for potential confounders.

#### Aim 4. Identifying the top reported item level determinants associated with program delivery duration from domains significantly associated with delivery duration (sustainment)

To identify the most influential item level determinants, each of the 28 items of the sustainment determinants measure were ranked in order of their perceived influence. The ‘moderately influential’ and ‘extremely influential’ responses were combined. The number and percentage of principals reporting a ‘moderately/extremely influential’ score for each of the 28 items was calculated, presented with corresponding 95% confidence intervals (CI) and *p*-value, and ranked from highest to lowest. The top reported determinants to sustainment were identified as the items most frequently perceived as influential, from measure domains which were significantly associated with sustainment.

## Results

### School and participant characteristics

Of the 295 randomly selected primary schools invited to participate in this sustainment component of the survey, 88 schools were found to be ineligible and therefore excluded. The principal or a selected nominee for 207 (70.2% of the total eligible sample) schools participated in the study. More than half of the included schools (64.2%) were government funded, and 59.4% of participants had been in their role for between 12 months and five years. The characteristics of participating schools are shown in Table 1.

**Table 1:** Principal and school characteristics

Demographic characteristics	Total (n)	Eligible Australian schools (%)
Total schools	207	7,279
Physical activity programs	86 (41.5%)	-
Nutrition programs	121 (58.4%)	-
School type		
Government	133 (64.2%)	69.29
Catholic	47 (22.7%)	17.85
Independent	27 (13%)	12.85
School size (No. of enrolments)		
Small (< 300)	121 (58.4)	52.67
Large (> 300)	86 (41.5%)	47.33
Socioeconomic status		
Most disadvantaged	106 (51.2%)	52.83
Least disadvantaged	101 (48.8%)	47.17
Area		
Remote (inner regional, outer regional, remote)	105 (50.5%)	48.65
Urban (major city)	102 (49.5%)	51.35
Principal role type		
Principal	178 (86%)	-
Deputy/Assistant Principal	16 (7.7%)	-
Head of school/campus/operations/Lead classroom teacher/**S**chool counsellor/social worker	7 (3.4%)	-
Health and wellbeing teacher/coordinator	6 (2.9%)	-
Principal time in role		
< 12 months	33 (15.9%)	-
12 months – 5 years	123 (59.4%)	-
> 5 years	51 (24.6%)	-

### Prevalence of physical activity and nutrition program

Of the 121 schools randomised to answer questions related to nutrition programs, schools reported implementing a mean of 2.8 of the eight included nutrition programs per school (range 1–7; standard deviation [SD] = 1.3). Table 2 shows that the most prevalent nutrition programs were: fruit and vegetable breaks, food growing experiences, and free fruit and vegetables.

**Table 2:** Description of programs

Physical activity	Prevalence n (%)	Nutrition	Prevalence n (%)
Environments that support physical activity	84 (97.7%)	Fruit and vegetable breaks	117 (96.7%)
Short physical activity classroom breaks	71 (85.6%)	Food growing experiences	96 (79.4%)
Physical activity within other key learning areas	65 (75.6%)	Free fruit and vegetables	47 (38.8%)
Teacher-led physical activity at recess and lunch	57 (66.3%)	School breakfast programs	43 (35.5%)
Programs or strategies to increase the intensity or the quality of physical education	53 (61.6%)	Cooking program(s) with parents	16 (13.22%)
Student-led physical activity at recess and lunch	50 (58.1%)	Incentives for choosing healthy food	7 (5.8%)
Standing desks	35 (40.7%)	Healthy eating strategies for canteens	7 (5.8%)
Trackers to monitor students’ physical activity	3 (3.5%)		
Physical activity-enabling uniforms everyday	2 (2.3%)		

The 86 schools randomised to physical activity programs were implementing a mean of 5.4 of the nine included physical activity programs per school (range 1–8; SD = 1.6). The most prevalent physical activity programs were: physical activity within other key learning areas, short physical activity classroom breaks, and teacher-led physical activity at recess and lunch (Table 2).

### Duration of program delivery

The mean duration for the selected physical activity programs principals reported on was 6.9 years (SD = 7.3). In contrast, nutrition programs exhibited a slightly longer mean duration of 7.4 years (SD = 5.0), however this difference was not statistically significant (see Table 3).

**Table 3:** Differences in program duration between physical activity and nutrition programs using linear mixed regression analysis

Program Type	N	Mean (SD) (years)	Median (Q1, Q3)	Mean Difference	Regression coefficient (95% CI)	*p*-value
Total	207	7.2 (6.1)	5.00 (3.00, 10.00)			
Physical Activity	86	6.9 (7.3)	4.00 (2.00, 10.00)	0.53	0.37 (−1.33, 2.06)	0.67
Nutrition	121	7.4 (5.0)	6.00 (4.00, 10.00)			

*SD* Standard Deviation, *CI* Confidence Interval, Statistical significance set at *P* = 0.05, Confounders = school type (government/Catholic), jurisdiction type, education sector, rurality (very remote, remote, outer regional, inner regional, major cities), SES, and school size

### Sustainment of PA vs nutrition programs

A statistically significant relationship was observed between sustainment duration and physical activity program (used as the reference category) and nutrition program. Nutrition programs had 3.27 times the odds of being sustained longer compared to physical activity programs (Table 4).

**Table 4:** Differences between phases of sustainment between physical activity and nutrition programs using mixed ordinal logistic regression analysis

Category of sustainment	Physical activity programs (N)	Nutrition programs (N)	Difference (Odds Ratio) and CI	*P* value	Proportional odds *p*-value
Not yet sustained	3	3	3.27 (1.57, 6.83)	0.002	< 0.001*
Early maintenance (6–12 months post implementation)	4	2			
Maintenance (12–24 months post implementation)	21	9			
Sustained/ing (24 + months post implementation)	58	107			

^*^Statistical significance with significance set at *P* = 0.05, *CI *Confidence interval. Confounders = school type (government/Catholic), jurisdiction type, education sector, rurality (very remote, remote, outer regional, inner regional, major cities), SES, and school size

### Association between measure domains and program sustainment

Outer Contextual Factors had a mean domain score of 3.78 out of 5.00. Principals who rated items from this domain as influential overall across both physical activity and nutrition programs reported statistically significantly longer durations of program delivery averaging 1.43 years longer. The other domains showed positive but non-statistically significant associations with program duration: Inner contextual factors; Processes; Characteristics of the intervention Table 5.

**Table 5:** Relationships between measure domains and duration of program delivery using linear mixed regression analysis

Measure domain	Mean domain score	Mean (SD) delivery time for those who selected item as being influential	Regression estimate	CI (95%)	*P*-Value	ICC
Outer contextual factors	3.78	7.57 (6.54)	1.4	(0.04, 2.77)	0.04*	0.00
Inner contextual factors	4.03	6.94 (6.00)	0.17	(−1.06, 1.40)	0.79	0.00
Processes	3.68	7.18 (6.18)	0.83	(−0.41, 2.08)	0.19	0.06
Characteristics of the intervention	4.17	7.24 (6.17)	1.06	(−0.45, 2.56)	0.17	0.00

*CI* Confidence interval

^*^Statistically significant with significance set at 0.05, Confounders = school type (government/Catholic), jurisdiction type, education sector, rurality (very remote, remote, outer regional, inner regional, major cities), SES, and school size

### Determinants influencing the sustainment of physical activity/nutrition programs

Table 6 shows the ranking of the three item level determinants influencing sustainment from the domain associated with longer delivery time (outer contextual factors). These determinants were: the alignment of the program with the priorities of the school, partnerships between the school and external organisations, and the existence of a governing body policy or guideline related to the program. For both physical activity and nutrition, the highest ranked determinant was the alignment of the program with the priorities of the school, followed by partnerships between the school and external organisations, and lastly, the existence of a governing body policy or guideline related to the program.

**Table 6:** Ranking of items from the outer contextual factors domain

Physical activity programs			Nutrition programs		
Measure Item	Number of respondents (n)	% principals who agreed the item was influential (n)	Measure Item	Number of respondents (n)	% principals who agreed the item was influential (n)
Alignment of the program with the priorities of my school	77	68.8 (53)	Alignment of the program with the priorities of my school	113	71.7 (81)
Partnerships between my school and external organisations	71	57.8 (41)	Partnerships between my school and external organisations	94	53.2 (50)
A governing body policy or guideline related to the program at my school	65	38.5 (25)	A governing body policy or guideline related to the program at my school	90	51.1 (46)

When completing the survey, ‘the program’ was replaced with the specific program which principals were individually randomised to

## Discussion

This is the first study to examine the prevalence and duration of the sustained delivery of physical activity and nutrition programs in Australian primary schools, and to explore the key determinants influencing program sustainment and their association with delivery duration. Outer contextual factors was the only domain which was significantly associated with greater program sustainment. The item level determinants within this domain includes: the alignment of the program with the priorities of the school, partnerships between the school and external organisations, and the existence of a governing body policy or guideline related to the program. The highest ranked determinant from this domain for both physical activity and nutrition was the alignment of the program with the priorities of the school. Developing an accurate and comprehensive understanding of sustainability determinants is important to inform the development of strategies to support the sustained delivery of such programs within this setting.

Our study provides insight into the prevalence of physical activity and nutrition programs which systematic reviews have identified as potentially effective [11, 12, 26, 27]. The schools answering in relation to physical activity programs reported implementing an average of 5.4 of the nine included physical activity programs per school. With the most prevalent being “physical activity within other key learning areas”, “short physical activity classroom breaks”, and “teacher-led physical activity at recess and lunch”. Schools answering in relation to nutrition programs, reported implementing an average of 2.8 of the eight included nutrition programs per school, with the most prevalent being “fruit and vegetable breaks”, “food growing experiences”, and “free fruit and vegetables”. This highlights the growing importance schools place on the promotion of healthy behaviours among school-aged children. These findings are consistent with previous research, such as the study conducted in Australian primary schools between 2006 and 2013, which observed significant improvements in the adoption of health policies and practices over time [23]. This suggests that there is now an increased awareness from school staff that school-based health programs are crucial for improving health outcomes among students. However, our data shows that some programs have low levels of adoption, implementation, and sustainment, specifically: Physical activity-enabling uniforms every day, and Healthy eating strategies for canteens. One plausible reason for the low adoption, implementation, and sustainment of these programs may be that they require more significant and systemic change to school operations and therefore need more support from school principals and executives. A recent study which explored primary school principals’, teachers’, and parents’ attitudes to changing school uniform policies to allow students to wear physical activity-enabling uniforms every day, found that the majority of principals were not supportive of such a change and that strategies to improve principal support may be required if broader adoption, implementation, and sustainment of this program is to be achieved [35]. Further, systematic review evidence regarding the barries to the implementation of, and compliance with school-based healthy food and beverage policies found that financial (e.g., cost of healthy foods) physical (e.g., availability of healthy foods) and social (stakeholders’ attitudes towards healthy eating policies) factors were the most frequently reported barriers for policy implementation [36]. Strategies aimed at addressing these barriers may be warranted to improve the adoption, implementation, and sustainment of programs such as healthy eating strategies for school canteens.

While there was very little difference in the mean duration of physical activity and nutrition programs (approximately 7 months), further analysis found a significant difference in sustainment categories by program type, with nutrition programs having approximately 3.27 times the odds of being sustained longer compared to physical activity programs (95% CI: 1.57, 6.83, *p* = 0.002). While further research is warranted to explore the factors contributing to this discrepancy, one plausible explanation could be the relative ease of the listed nutrition programs compared to the physical activity programs in terms of their teacher and resource requirements. Unlike physical activity programs, which often require specialised equipment, designated spaces, and trained staff for implementation [37], the most prevalent pre-specified nutrition programs can mostly be delivered with minimal time, skill, infrastructure, and resources [13]. This reduced burden may make some simple nutrition programs e.g., fruit and vegetable breaks where children consume a piece of vegetable or fruit in class that they have brought from home [11] more amenable to sustainment, particularly in resource-constrained environments. Additionally, the inherent flexibility of nutrition interventions, which can be adapted to accommodate cultural preferences, dietary habits, and socioeconomic contexts, may also contribute to their sustainability [38]. As a result, more intensive sustainment support may be needed to assist in the long-term delivery of physical activity programs compared to nutrition programs in schools. However, further research is needed to unpack the nuanced mechanisms driving their sustainability.

We found that outer contextual factors, which includes things such as: external funding, external leadership support, alignment with organisational values, needs, and priorities, and the socio-political context of program implementation, was the only domain significantly associated with prolonged program sustainment. This is consistent with prior research [18, 19], which highlights the critical role that these factors play in sustaining programs in schools. Further, a longitudinal study on the sustainment of the Adolescent-Community Reinforcement Approach (A-CRA) found that the lack of outer contextual factor level support and resources, such as consistent funding and leadership support, significantly hindered the sustainability of the program [39]. This emphasises a hierarchical interplay between determinants, suggesting a need to prioritise addressing outer contextual factors in schools. From this domain, we found that principals perceived the alignment of the program with the priorities of the school to be the most influential determinant to the sustainability for both physical activity and nutrition programs. Future efforts to enhance the sustainability of school-based health programs should focus on developing targeted strategies to enhance the fit of programs to the needs of the school. Strategies could include taking a co-creation approach during the initial stages of program development. Including and engaging with key stakeholders, such as school executives, teachers, and parents, in the planning process may help to ensure programs address specific school priorities. Regular monitoring and adjusting of the program to align with evolving school goals and student needs may also be needed to ensure long term sustainment. However, research is still scarce on the effectiveness of sustainment strategies [21]. A recent review found only three included studies tested the effectiveness of strategies designed to support sustainment, none of which were conducted in the school setting [31]. This highlights the need for further research to empirically explore the effectiveness of sustainment strategies to sustain the delivery of school-based health programs [31].

### Limitations

The findings of this study must be considered in the context of its limitations. First, although we followed robust methods for developing the measure of sustainability determinants, we were unable to conduct a complete psychometric evaluation due to a low sample size, therefore only internal consistency was assessed. Secondly, the data used in this study was cross-sectional and did not look at determinants to sustainment over time. Third, there may be different determinants that are deemed most influential for different priority populations (e.g., schools with a high proportion of Aboriginality, low socioeconomic schools, and rurality), therefore warrants additional investigation in these population groups. Fourth, we included a finite number of practices, and principals only completed the survey based on a single program. It is possible that we have missed important information regarding the sustainment of programs not included in our study and that we have gained a deeper understanding if principals reported on all of their programs. Further, as principals only reported on the determinants of a single program, results may vary depending on which program they report on. Finally, although we provide insight into the prevalence of physical activity and nutrition programs, this prevalence was calculated after schools had already been randomised into the physical activity arm or nutrition arm. To gain a better understanding of the prevalence of these programs, future research should assess the overall collective prevalence of these programs.

## Conclusion

This study highlights the main determinants influencing the sustainability of physical activity and nutrition programs in schools. These data which can be used by policymakers and practitioners to help support sustainment of such programs. Tailoring sustainment strategies to address the distinct challenges faced by each health programs is essential for sustainment. It is evident that determinants related to outer contextual factors are of key importance, specifically, the alignment of the program with the priorities of the school. Prioritising the development of strategies aimed at these outer contextual factors is crucial to effectively support program sustainment and impact long-term health outcomes.

## Supplementary Information

Supplementary Material 1.

Supplementary Material 2.

## Data Availability

Data and materials relating to this study are available from the corresponding author on reasonable request.

## Abbreviations

SES: Socioeconomic status
REDCap: Research Electronic Data Capture
CATI: Computer Assisted Telephone Interview
