# Supplementary material for: Identifying key determinants influencing the sustainment of physical activity and nutrition programs in Australian primary schools
Source: Int J Behav Nutr Phys Act. 2025 Aug 30;22:116. doi: 10.1186/s12966-025-01808-6 (PMC12399001; doi:10.1186/s12966-025-01808-6)
Supplement: Supplementary file 1 — Supplementary Material 1. [file 12966_2025_1808_MOESM1_ESM.docx]

Appendix 1. NCOIS School Principal Sustainability Survey


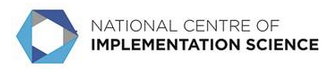


**NCOIS School Principal Survey of CDP Practice Implementation**

Version 7, dated 13/04/2022

**Sample =1 Nutrition**

**Sample =2 PA**

**Sample =3 Tob/Alc**

**MODULE 8: Intervention Sustainability Module**

******RANDOMISATION 3****** [split_kt_m8]='2'

**Each participant will be randomly allocated to x1 healthy eating or x1 physical activity practices they reported implementing earlier in the survey**

| INFO8 if not pa3 or pa5 or pa6 or pa19 | We would like to know what factors influence the ongoing delivery of health programs in schools.  For the next section of the survey, we would like to ask you about the [programlabel] you reported earlier in the survey your school currently implements. By this we mean **[**programdef**].** | Go to S1 |
| --- | --- | --- |

| INFO8_1 | We would like to know what factors influence the ongoing delivery of health programs in schools.  You reported earlier in the survey that your school currently implements **[**pipe open-ended response], the definition of which is **[**programdef].  For the next section of the survey, we would like you to choose one strategy that you implement. | |
| --- | --- | --- |
|  | Enter text here | Go to S1 |

| S1 | | Approximately how long has your school been delivering [programlabel] (in years or months) | |
| --- | --- | --- | --- |
|  |  | 1 Years (enter number is years, enter numerical value) | Go to S2 |
|  |  | 2 Months (if less than 1 year, enter numerical value 1-11) |  |
|  |  | 3 Less than 1 month |  |
|  |  | 888, Unsure |  |
|  |  | 999, Prefer not to say |  |

| INFO8a | The following questions will ask you about the extent to which you believe a range of factors influence the continued delivery of [programlabel] at your school.  Please answer each question based on your experience in delivering [programlabel]. There are no right or wrong answers.  Please choose a response option that most closely matches how influential you believe the following factors are on your delivery of [programlabel].  If any of the statements are not applicable to you (e.g., does not exist in your school), please choose the “Not applicable to me” option.  We will ask you to answer each question using a scale from not applicable to me, to extremely influential. Where:  1 = Not applicable to me. This factor does not exist or apply to your school;  2 = Not at all influential. This factor exists within your school, but it is not at all influential in your school’s ability to deliver the program;  3 = Slightly influential. This factor exists within your school and has only a small influence on your school’s ability to deliver the program;  4 = Moderately influential. This factor exists within your school and has a moderate influence on your school’s ability to deliver the program; and  5 = Extremely influential. This factor exists within your school and your school could not deliver the program without this factor. |
| --- | --- |

| S2 O | | A governing body policy or guideline related to [programlabel] at my school.  ***NOTE:*** *A governing body refers to an educational department or authority e.g., NSW Department of Education* | |
| --- | --- | --- | --- |
|  |  | 1 Not applicable to me | Go to S3 |
|  |  | 2 Not at all influential |  |
|  |  | 3 Slightly influential |  |
|  |  | 4 Moderately influential |  |
|  |  | 5 Extremely influential |  |
|  |  | 888, Unsure |  |
|  |  | 999, Prefer not to say |  |

| S3 O | | Partnerships between my school and external organisations.  ***NOTE****: Examples of partnerships could include government agencies, councils and health organisations* | |
| --- | --- | --- | --- |
|  |  | 1 Not applicable to me | Go to S4 |
|  |  | 2 Not at all influential |  |
|  |  | 3 Slightly influential |  |
|  |  | 4 Moderately influential |  |
|  |  | 5 Extremely influential |  |
|  |  | 888, Unsure |  |
|  |  | 999, Prefer not to say |  |

| S4 O | | Alignment of [programlabel] with the priorities of my school. | |
| --- | --- | --- | --- |
|  |  | 1 Not applicable to me | Go to S5 |
|  |  | 2 Not at all influential |  |
|  |  | 3 Slightly influential |  |
|  |  | 4 Moderately influential |  |
|  |  | 5 Extremely influential |  |
|  |  | 888, Unsure |  |
|  |  | 999, Prefer not to say |  |

| S5 | | Program champions at my school.  ***NOTE****: a champion is a colleague chosen to drive the delivery of the program at your school* | |
| --- | --- | --- | --- |
|  |  | 1 Not applicable to me | Go to S6 |
|  |  | 2 Not at all influential |  |
|  |  | 3 Slightly influential |  |
|  |  | 4 Moderately influential |  |
|  |  | 5 Extremely influential |  |
|  |  | 888, Unsure |  |
|  |  | 999, Prefer not to say |  |

| S6 | | The overall support from school executives. | |
| --- | --- | --- | --- |
|  |  | 1 Not applicable to me | Go to S7 |
|  |  | 2 Not at all influential |  |
|  |  | 3 Slightly influential |  |
|  |  | 4 Moderately influential |  |
|  |  | 5 Extremely influential |  |
|  |  | 888, Unsure |  |
|  |  | 999, Prefer not to say |  |

| S7 | | Support from school executives for staff to access training to deliver [programlabel]. | |
| --- | --- | --- | --- |
|  |  | 1 Not applicable to me | Go to S8 |
|  |  | 2 Not at all influential |  |
|  |  | 3 Slightly influential |  |
|  |  | 4 Moderately influential |  |
|  |  | 5 Extremely influential |  |
|  |  | 888, Unsure |  |
|  |  | 999, Prefer not to say |  |

| S8 | | The physical space available for staff to deliver [programlabel]. | |
| --- | --- | --- | --- |
|  |  | 1 Not applicable to me | Go to S9 |
|  |  | 2 Not at all influential |  |
|  |  | 3 Slightly influential |  |
|  |  | 4 Moderately influential |  |
|  |  | 5 Extremely influential |  |
|  |  | 888, Unsure |  |
|  |  | 999, Prefer not to say |  |

| S9 | | The equipment available for staff to deliver [programlabel]. | |
| --- | --- | --- | --- |
|  |  | 1 Not applicable to me | Go to S10 |
|  |  | 2 Not at all influential |  |
|  |  | 3 Slightly influential |  |
|  |  | 4 Moderately influential |  |
|  |  | 5 Extremely influential |  |
|  |  | 888, Unsure |  |
|  |  | 999, Prefer not to say |  |

| S10 | | The funding available for staff to deliver [programlabel]. | |
| --- | --- | --- | --- |
|  |  | 1 Not applicable to me | Go to S11 |
|  |  | 2 Not at all influential |  |
|  |  | 3 Slightly influential |  |
|  |  | 4 Moderately influential |  |
|  |  | 5 Extremely influential |  |
|  |  | 888, Unsure |  |
|  |  | 999, Prefer not to say |  |

| S11 | | The time available for staff during the school day to deliver [programlabel]. | |
| --- | --- | --- | --- |
|  |  | 1 Not applicable to me | Go to S12 |
|  |  | 2 Not at all influential |  |
|  |  | 3 Slightly influential |  |
|  |  | 4 Moderately influential |  |
|  |  | 5 Extremely influential |  |
|  |  | 888, Unsure |  |
|  |  | 999, Prefer not to say |  |

| S12 | | Staff feeling prepared to deliver [programlabel] if there is a change of executive at my school. | |
| --- | --- | --- | --- |
|  |  | 1 Not applicable to me | Go to S13 |
|  |  | 2 Not at all influential |  |
|  |  | 3 Slightly influential |  |
|  |  | 4 Moderately influential |  |
|  |  | 5 Extremely influential |  |
|  |  | 888, Unsure |  |
|  |  | 999, Prefer not to say |  |

| S13 | | Staff feeling supported to deliver [programlabel] if there are changes to staff members at my school. | |
| --- | --- | --- | --- |
|  |  | 1 Not applicable to me | Go to S14 |
|  |  | 2 Not at all influential |  |
|  |  | 3 Slightly influential |  |
|  |  | 4 Moderately influential |  |
|  |  | 5 Extremely influential |  |
|  |  | 888, Unsure |  |
|  |  | 999, Prefer not to say |  |

| S14 | | The training received by staff to deliver [programlabel].  ***NOTE:*** *Training may include in house or external workshops or courses* | |
| --- | --- | --- | --- |
|  |  | 1 Not applicable to me | Go to S15 |
|  |  | 2 Not at all influential |  |
|  |  | 3 Slightly influential |  |
|  |  | 4 Moderately influential |  |
|  |  | 5 Extremely influential |  |
|  |  | 888, Unsure |  |
|  |  | 999, Prefer not to say |  |

| S15 | | The feedback staff receive regarding their delivery of [programlabel].  ***NOTE****: Feedback may be from peers or program co-ordinators, or student or parent surveys* | |
| --- | --- | --- | --- |
|  |  | 1 Not applicable to me | Go to S16 |
|  |  | 2 Not at all influential |  |
|  |  | 3 Slightly influential |  |
|  |  | 4 Moderately influential |  |
|  |  | 5 Extremely influential |  |
|  |  | 888, Unsure |  |
|  |  | 999, Prefer not to say |  |

| S16 | | My school’s ability to adapt [programlabel] based on routine evaluation of how well [programlabel] fits with our priorities. | |
| --- | --- | --- | --- |
|  |  | 1 Not applicable to me | Go to S17 |
|  |  | 2 Not at all influential |  |
|  |  | 3 Slightly influential |  |
|  |  | 4 Moderately influential |  |
|  |  | 5 Extremely influential |  |
|  |  | 888, Unsure |  |
|  |  | 999, Prefer not to say |  |

| S17 | | My school’s documented plan for how staff should deliver [programlabel] long-term. | |
| --- | --- | --- | --- |
|  |  | 1 Not applicable to me | Go to S18 |
|  |  | 2 Not at all influential |  |
|  |  | 3 Slightly influential |  |
|  |  | 4 Moderately influential |  |
|  |  | 5 Extremely influential |  |
|  |  | 888, Unsure |  |
|  |  | 999, Prefer not to say |  |

| S18 | | The communication between my school and the wider community about our plan to deliver [programlabel] long term.  ***NOTE****: school community refers to administrators, teachers, staff members, children, their parents/guardians and families directly involved with your school* | |
| --- | --- | --- | --- |
|  |  | 1 Not applicable to me | Go to S19 |
|  |  | 2 Not at all influential |  |
|  |  | 3 Slightly influential |  |
|  |  | 4 Moderately influential |  |
|  |  | 5 Extremely influential |  |
|  |  | 888, Unsure |  |
|  |  | 999, Prefer not to say |  |

| S19 | | My schools’ ability to adapt [programlabel] to match the resources/equipment available. | |
| --- | --- | --- | --- |
|  |  | 1 Not applicable to me | Go to S20 |
|  |  | 2 Not at all influential |  |
|  |  | 3 Slightly influential |  |
|  |  | 4 Moderately influential |  |
|  |  | 5 Extremely influential |  |
|  |  | 888, Unsure |  |
|  |  | 999, Prefer not to say |  |

| S20 | | My school’s ability to adapt [programlabel] to suit the physical school environment. | |
| --- | --- | --- | --- |
|  |  | 1 Not applicable to me | Go to S21 |
|  |  | 2 Not at all influential |  |
|  |  | 3 Slightly influential |  |
|  |  | 4 Moderately influential |  |
|  |  | 5 Extremely influential |  |
|  |  | 888, Unsure |  |
|  |  | 999, Prefer not to say |  |

| S21 | | My school’s ability to adapt [programlabel] to fit within our regular school schedule. | |
| --- | --- | --- | --- |
|  |  | 1 Not applicable to me | Go to S22 |
|  |  | 2 Not at all influential |  |
|  |  | 3 Slightly influential |  |
|  |  | 4 Moderately influential |  |
|  |  | 5 Extremely influential |  |
|  |  | 888, Unsure |  |
|  |  | 999, Prefer not to say |  |

| S22 | | The appropriateness of [programlabel] for all children at my school, regardless of their socioeconomic background. | |
| --- | --- | --- | --- |
|  |  | 1 Not applicable to me | Go to S23 |
|  |  | 2 Not at all influential |  |
|  |  | 3 Slightly influential |  |
|  |  | 4 Moderately influential |  |
|  |  | 5 Extremely influential |  |
|  |  | 888, Unsure |  |
|  |  | 999, Prefer not to say |  |

| S23 | | The cultural appropriateness of [programlabel] for all children at my school. | |
| --- | --- | --- | --- |
|  |  | 1 Not applicable to me | Go to S24 |
|  |  | 2 Not at all influential |  |
|  |  | 3 Slightly influential |  |
|  |  | 4 Moderately influential |  |
|  |  | 5 Extremely influential |  |
|  |  | 888, Unsure |  |
|  |  | 999, Prefer not to say |  |

| S24 | | Knowing [programlabel] was developed by a reputable organisation. | |
| --- | --- | --- | --- |
|  |  | 1 Not applicable to me | Go to S25 |
|  |  | 2 Not at all influential |  |
|  |  | 3 Slightly influential |  |
|  |  | 4 Moderately influential |  |
|  |  | 5 Extremely influential |  |
|  |  | 888, Unsure |  |
|  |  | 999, Prefer not to say |  |

| S25 | | [programlabel] being widely accepted by teachers at my school. | |
| --- | --- | --- | --- |
|  |  | 1 Not applicable to me | Go to S26 |
|  |  | 2 Not at all influential |  |
|  |  | 3 Slightly influential |  |
|  |  | 4 Moderately influential |  |
|  |  | 5 Extremely influential |  |
|  |  | 888, Unsure |  |
|  |  | 999, Prefer not to say |  |

| S26 | | [programlabel] being easily delivered at my school. | |
| --- | --- | --- | --- |
|  |  | 1 Not applicable to me | Go to S27 |
|  |  | 2 Not at all influential |  |
|  |  | 3 Slightly influential |  |
|  |  | 4 Moderately influential |  |
|  |  | 5 Extremely influential |  |
|  |  | 888, Unsure |  |
|  |  | 999, Prefer not to say |  |

| S27 | | The health benefits of [programlabel] for children at my school. | |
| --- | --- | --- | --- |
|  |  | 1 Not applicable to me | Go to S28 |
|  |  | 2 Not at all influential |  |
|  |  | 3 Slightly influential |  |
|  |  | 4 Moderately influential |  |
|  |  | 5 Extremely influential |  |
|  |  | 888, Unsure |  |
|  |  | 999, Prefer not to say |  |

| S28 | | The cost to deliver [programlabel] at my school being acceptable. | |
| --- | --- | --- | --- |
|  |  | 1 Not applicable to me | Go to S29 |
|  |  | 2 Not at all influential |  |
|  |  | 3 Slightly influential |  |
|  |  | 4 Moderately influential |  |
|  |  | 5 Extremely influential |  |
|  |  | 888, Unsure |  |
|  |  | 999, Prefer not to say |  |

| S29 | | My belief that delivering [programlabel] is as important as other learning areas at my school. | |
| --- | --- | --- | --- |
|  |  | 1 Not applicable to me | Go to RESULTS |
|  |  | 2 Not at all influential |  |
|  |  | 3 Slightly influential |  |
|  |  | 4 Moderately influential |  |
|  |  | 5 Extremely influential |  |
|  |  | 888, Unsure |  |
|  |  | 999, Prefer not to say |  |

Appendix 2**:** Ethics approvals

| **Jurisdiction** | **Approval number** |
| --- | --- |
| ACT Department of Education | RES 2314 |
| ACT/NSW Archdiocese of Canberra and Goulburn | NA |
| NSW Department of Education (SERAP) | 2021187 |
| Diocese of Bathurst | NA |
| Diocese of Maitland-Newcastle | NA |
| Diocese of Parramatta | NA |
| Diocese of Wagga Wagga | NA |
| NT Department of Education | 20817 |
| NT Catholic schools | NA |
| QLD Department of Education | 550/27/2550 |
| Archdiocese of Brisbane | 502 |
| Diocese of Cairns | NA |
| Diocese of Rockhampton | NA |
| Diocese of Townsville | 2021-13 |
| SA Department of Education | 2021-0074 |
| SA Catholic schools | 202134 |
| TAS Department of Education | 2022-03 |
| Archdiocese of Hobart | NA |
| VIC Department of Education | 2022_004563 |
| Archdiocese of Melbourne | 1173 |
| Diocese of Ballarat | NA |
| Diocese of Sale | NA |
| Diocese of Sandhurst | NA |
| WA Department of Education | D23/1411981 |
| Catholic Education Western Australia (four Dioceses (Broome, Bunbury, Geraldton, Perth) | RP2021/44 |

ACT: Australian Capital Territory; QLD: Queensland; NA: Not Applicable; NSW: New South Wales; NT: Northern Territory; SA: South Australia; SD: standard deviation; SES: Socio-Economic Status; SERAP: State Education Research Application Process; TAS: Tasmania; VIC: Victoria; WA: Western Australia
